# Isocratic Resolution of Fluoroquinolone-Based Antibiotics on the Phenylethyl-Bonded Phase under Nonaqueous Elution: A Consideration of the Separation Mechanism

**DOI:** 10.1155/2018/1375215

**Published:** 2018-05-31

**Authors:** Yu-Xuan Gao, Shushi Chen

**Affiliations:** Department of Applied Chemistry, National Chiayi University, Chiayi 600, Taiwan

## Abstract

This paper reports the isocratic resolution of 10 fluoroquinolone-based antibiotics and their precursors on the phenylethyl-bonded phase under the elution of the nonaqueous mobile phase composed of acetonitrile, methanol, acetic acid, and triethylamine. Most of the analytes were baseline resolved within 10 minutes. The interaction simulation and Fourier-transform infrared spectroscopy (FTIR) data indicated that the carbonyl-containing group, a secondary or tertiary amine of an analyte, was heavily involved in the retention, resulting in retention with residual silanol groups on the stationary phase. In some cases, the elution reversal or resolution enhancement of analytes was observed when the volume of acidic or basic additive in the mobile phase was dominant. However, the *π*-*π* complexation interaction between the fluorine-attached phenyl group of the analyte and the phenylethyl moiety on the stationary phase was not observed. Consequently, the resolution could not be reproduced either on the other stationary phase modified with C_18_, phenyl, or phenylhexyl moiety under the same chromatographic conditions or under the aqueous elution.

## 1. Introduction

Quinolone-based antibiotics are universally effective and used extensively against Gram-negative bacteria in livestock and humans to treat a wide variety of diseases [1–4]. Because fluoroquinolones and their precursors are only partially metabolized after administration, these compounds are discharged into municipal wastewater and environmental aquatic systems. Antibiotic pollution in agricultural crops is expected if the crops are irrigated with these water resources and fertilized with sewage sludge from water treatment plants or livestock manure. For environmental and public safety, several methods have been developed for high-performance liquid chromatography (HPLC) analysis and the recovery of these antibiotics from wastewater and soil using adsorbents [5–17]. The HPLC approach in these studies often involved gradient or isocratic elution combined with a C_18_ stationary phase to resolve a limited number of antibiotics. However, an inconsistent elution order is often observed under the aqueous chromatographic condition [10, 15–17].

In this study, several stationary phases, including phenyl (C_ph_), phenylethyl (C_phe_), phenylhexyl (C_phh_), C_8_, and C_18_ were examined to optimize the resolution of 10 fluoroquinolone-based antibiotics and their precursors under isocratic nonaqueous elution. The resolution was further optimized by altering the volume of acidic or basic additive in the mobile phase. Under optimized conditions, Fourier-transform infrared spectroscopy (FTIR) data were collected to explore the resolution mechanism. In addition, interaction simulation was performed to gain a theoretical understanding of the mechanism leading to the resolution.

## 2. Experimental Procedures

### 2.1. Apparatus

An HPLC system (Model L-7100, Tokyo, Japan) coupled with a D-2500 chromatopac data station (Shimadzu, Kyoto, Japan) and ultraviolet (UV) detector with the detection wavelength set at 294 nm was used for resolution optimization under nonaqueous elution. Columns packed with C_8_-, C_18_-, phenyl-, phenylethyl-, and phenylhexyl-modified silica gels (250 × 4.6 mm internal diameter; 5 *μ*m particle diameter) were manufactured by GL Sciences Inc. (Tokyo, Japan) and used for HPLC resolution at a flow rate of 1.0 mL/min. The mobile phase for HPLC elution was a mixture of acetonitrile, methanol, glacial acetic acid, and triethylamine.

FTIR spectra were obtained by scanning samples 10 times on a Shimadzu Model FTIR-8400 system at a resolution of 4 cm^−1^. In the FTIR measurements, a small fraction of the stationary phase in the column purchased was sampled and immersed in the mobile phase containing the analyte and then collected, pelleted with KBr after being washed with purified water, and dried.

### 2.2. Chemicals

All chemicals used in this study, including the fluoroquinolone-based antibiotics and their precursors, were purchased from Sigma (St. Louis, MO, USA) or Aldrich (Milwaukee, WI, USA). All HPLC-grade solvents (acetonitrile, methanol, glacial acetic acid, and triethylamine) were obtained from Fisher Scientific (Pittsburgh, PA, USA) and Merck Taiwan Ltd. (Taipei, Taiwan, ROC). In all cases, filtered (0.2 mm) and distilled water was used. Antibiotic standards were purchased to identify the chromatographic peaks.

### 2.3. Theoretical Computational Calculation with Spartan'14 Software

A theoretical calculation for single point energy was conducted according to a semiempirical molecular orbital calculation method (Parameterized Model 3) by using Spartan'14 software from Wavefunction, Inc. (Irvine, CA, USA). Atoms on the stationary phase and analyte were simulated to interact with one another to determine the lowest formation energy at the ground state (i.e., the heat of formation). Stationary phase moieties including C_8_, C_18_, phenyl, phenylethyl, and phenylhexyl placed around the fixed position analyte were considered in the evaluation. Prior to the calculation, the molecular energy was first minimized by modifying the bond lengths and angles until a minimum-energy conformer was found. The mutual distance between the analyte and stationary phase moiety was subsequently altered as a result.

## 3. Results and Discussion

### 3.1. Stationary Phase Consideration

**Figure 1:**
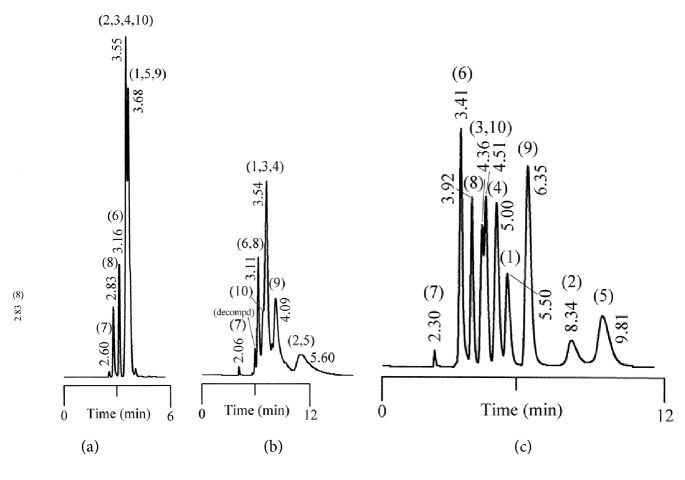
Isocratic resolution of 10 fluoroquinolone-based antibiotics and their precursors on the C_8_- (a), C_18_- (b), and phenylethyl-bonded stationary phases under optimized elution of nonaqueous mobile phases of 490/10/1/1, 500/0/1/3, and 490/10/1/2 by volume (acetonitrile/methanol/acetic acid/triethylamine, *v*/*v*), respectively. The numbering system of compounds from left to right is as follows: piroxicam (7), nalidixic acid (6), cinoxacin (8), difloxacin (3), enrofloxacin (10), pefloxacin (4), ofloxacin (1), lomefloxacin (9), ciprofloxacin (2), and pipemidic acid (5).

**Figure 2:**
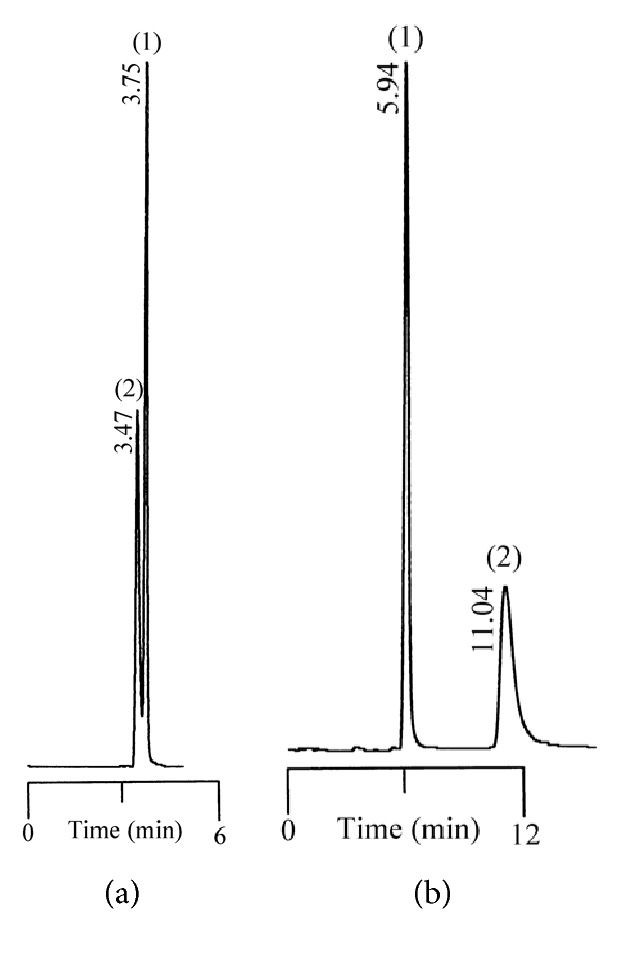
Resolution of ofloxacin and ciprofloxacin under the elution of ACN/1/3 by volume (acetonitrile/acetic acid/triethylamine, *v*/*v*) on the C_8_ (a) and phenylethyl (b) stationary phases. Note that the elution order has been reversed. The numbering system is the same as that in Figure 1.

Tables 1 and 2 summarize the chromatographic data for the resolution of nine selected fluoroquinolone-based antibiotics and their precursors on the phenylethyl-bonded phase under the isocratic elution of various nonaqueous mobile phases with the predominantly acidic or basic additive by volume, respectively. A typical chromatogram showing the resolution of 10 analytes under optimized conditions on the phenylethyl-boned stationary phase within 10 minutes is shown in Figure 1(c). Under the same conditions, the resolution was not reproducible on stationary phases such as C_8_ (a) and C_18_ (b) after comparison. Evidently, analytes were less retained and poorly resolved on the hydrocarbon-typed stationary phases, and the peak tailing for certain analytes strongly suggests that the hydrophobicity-oriented approach (e.g., C_8_ and C_18_ phases) toward the resolution is impractical. Upon close examination of these chromatograms, elution reversal for several analytes was observed on C_8_ but not on C_18_. This was evident in the resolution of ofloxacin and ciprofloxacin on C_8_ and C_phe_ phases under the elution of ACN/1/3 by volume (acetonitrile/acetic acid/triethylamine, *v*/*v*), as shown in Figure 2. The discrepancies in the chromatographic profile and elution order results strongly suggest that the hydrophobic interaction was not the only force responsible for resolution on the C_phe_ phase.

**Figure 3:**
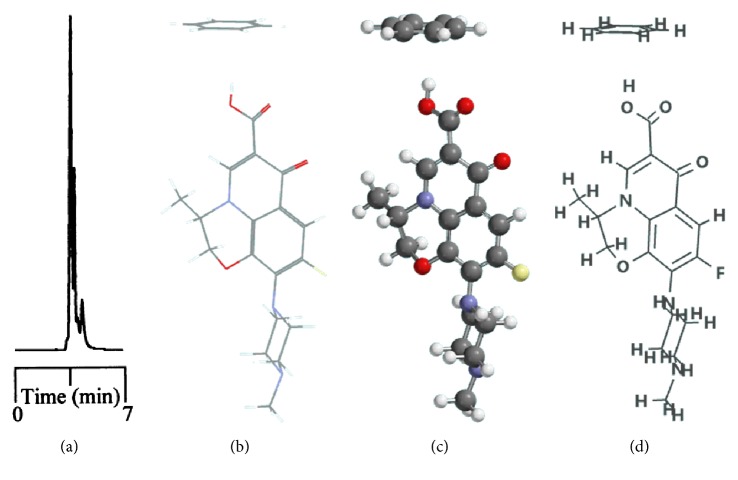
Resolution of 10 fluoroquinolone-based antibiotics and their precursors on the phenyl-bonded stationary phase under the elution of nonaqueous mobile phase of 490/10/1/2 by volume (acetonitrile/methanol/acetic acid/triethylamine, *v*/*v*) (a). The interaction simulation between ofloxacin and phenyl moiety expressed in stereochemistry molecular (b) and stick and ball (c) models and molecular structure (d) for easy comparison.

**Figure 4:**
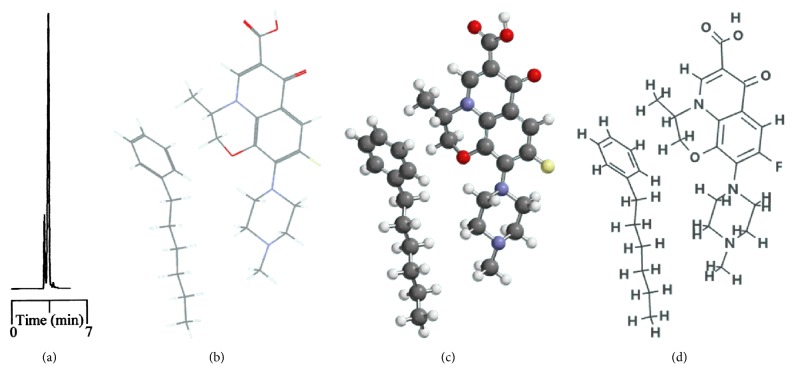
Resolution of 10 fluoroquinolone-based antibiotics and their precursors on the phenylhexyl-bonded stationary phase under the elution of nonaqueous mobile phase of 490/10/1/2 by volume (acetonitrile/methanol/acetic acid/triethylamine, *v*/*v*) (a). The interaction simulation between ofloxacin and phenylhexyl moiety expressed in stereochemistry molecular (b) and stick and ball (c) models and molecular structure (d) for easy comparison.

Phenyl- and phenylhexyl-modified silica gels were two other stationary phases examined in this study to further explore the involvement of *π*-*π* complexation in retention and, consequently, resolution under nonaqueous elution.  Figure 3(a) shows the resolution of analytes on the phenyl-bonded stationary phase under the same elution of the nonaqueous mobile phase of 490/10/1/2 by volume (acetonitrile/methanol/acetic acid/triethylamine, *v*/*v*). Notably, the only difference between the two stationary phases was in the alkyl group linking the aromatic moiety to the silica gel. However, the profile of the chromatogram, which was characterized by poor resolution and a short retention scale, was totally different from that in Figure 1(c) after comparison, indicating that the *π*-*π* complexation interaction was not the main force contributing to the resolution. In the interaction simulation between ofloxacin and the phenyl moiety (Figures 3(b), 3(c), and 3(d)), the same conclusion was reached based on a lack of observable *π*-*π* stacking complexation. Notably, these two conformers exhibited the most stable conformation among themselves and seven others (Table 3) when assessed based on the amount of energy released upon association. In the case of C_phh_, a phase possessing a long hydrocarbon linker, the resolution in Figure 4(a) showed no improvement under the same mobile phase elution. However, the hexyl hydrocarbon linker of the phase did contribute a degree of retention based on the simulation results in Figures 4(b), 4(c), and 4(d), similar to the C_8_ and C_18_ phases discussed previously. In addition, there was no evidence of *π*-*π* stacking complexation in this most stable conformation.

### 3.2. Interaction Simulation and FTIR Data

**Figure 5:**
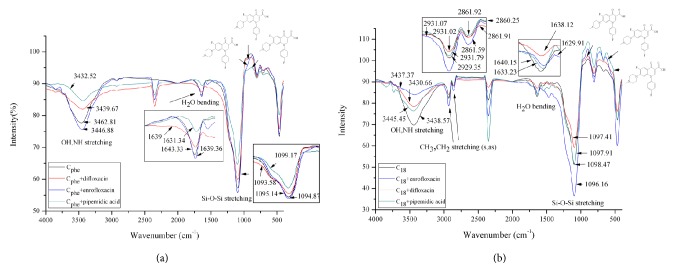
Superimposed FTIR spectra for three selected analytes, including difloxacin, enrofloxacin, and pipemidic acid on the phenylethyl (a) and C_18_ (b) phases. In both cases, FTIR spectra for phenylethyl and C_18_ phases were included for comparison.

Except in the case of ofloxacin, several analytes were examined in the interaction simulation with the three aforementioned stationary phases. The results listed in Table 3 indicate that generally the C_phh_ and C_ph_ phases released the most and least energy, respectively, from association with the analytes. Additionally, the number for the amount of energy released within the range specified in Table 3 was the greatest in the C_phh_ phase. All simulation results should lead to a significant retention scale because of the intensive interactions of two conformers and thus should lead to the possibility of improving the resolution on the C_phh_ phase. However, the resolution on these phases under the same chromatographic conditions was observed to be incomparable with that on the C_phe_ phase, strongly suggesting that there was some form of force other than hydrophobicity or *π*-*π* complexation interaction involved in the chromatographic process. Based on the FTIR data, residual silanol groups on the surface of the silica gel were considered responsible for producing the strong dipole-dipole interaction. Upon close examination of the spectra for three selected analytes with the C_phe_ phase in Figure 5(a), the stretching vibrations centered at 3463.72 cm^−1^ for the -OH or -NH functional group on the C_phe_ phase were all red shifted on a large scale because of the conformational interaction compared with that of the C_18_ phase (Figure 5(b)) under the same experimental conditions. Note that the acidity of both analyte and silanol group and thus the dipole-dipole interaction are enhanced in acetonitrile [18]. However, the enhancement would not be observed under the elution of aqueous mobile phase due to the competition interaction with silanol group from water molecule. Conversely, the shift of CH_3_ and CH_2_ stretching (symmetric and asymmetric) on the C_18_ phase was insignificant after association, indicating that the hydrophobic interaction between the analyte and C_18_ molecule was not the major force contributing to the retention on the phase. The peaks centered at 1640.15 and 1643.33 cm^−1^ in Figure 5 were assigned to the residual water molecule bending vibration on C_18_ and C_phe_ phases, respectively. The difference in bending frequency between these two phases was due to the environmental variation surrounding the water molecule. The environmental variation was further complicated as the analyte was near the phase molecule and interacting with it, which resulted in an apparent red shift in frequency. By contrast, the vibrational frequency of the Si-O-Si backbone network (centered at 1098.47 and 1095.14 cm^−1^ on C_18_ and C_phe_ phases, respectively) on both examined phases was not altered upon the association of the analyte and modifier molecule of the stationary phase. These FTIR results suggest that the interaction occurred near the surface, and thus the peak tailing that resulted from penetration of the analyte molecule deep into the silica matrix was not observed.

### 3.3. Effect of Acidic and Basic Additives on Retention, Elution Order, and Resolution

The chromatographic data in Tables 1 and 2 for the nine selected fluoroquinolone-based antibiotics and their precursors on phenylethyl-bonded phase under the isocratic elution of various nonaqueous mobile phases with predominantly acidic or basic additive by volume may provide experimental evidence of the interaction force contributing to resolution. Under the isocratic elution using the mobile phase with the predominantly acidic additive (Table 1), the capacity factor for all the examined analytes was increased with the volume of acidic additive. However, the selectivity factor could remain constant (e.g., pefloxacin versus ofloxacin) or become larger (e.g., difloxacin versus enrofloxacin) or smaller (e.g., ofloxacin versus lomefloxacin) in value, depending on how the capacity factor of the analyte is affected by the volume increment of acidic additive in the mobile phase. Notably, the resolution factor was enhanced considerably because of the improvement of the selectivity factor in the cases of difloxacin and enrofloxacin as the volume of acidic additive in the mobile phase was increased from 2 to 5 ml. In other words, difloxacin and enrofloxacin had much greater resolution values than those at baseline (*R*_*s*_ = 1.50) under the elution of the mobile phase 490/10/5/1 by volume (acetonitrile/methanol/acetic acid/triethylamine, *v*/*v*). Protonation of functional groups on the stationary phase and the analyte on a large scale enhanced the dipole-dipole interaction, and thus the magnitude of this effect was believed to be structure dependent. Essentially, this conclusion is consistent with many previous reports dealing with p*K*_a_ value determination for several quinolone and fluoroquinolone antibiotics in aqueous solutions [19–23].

The capacity factor for the resolution of all the examined analytes on the C_phe_ phase under isocratic elution using the mobile phase with the predominantly basic additive was decreased (Table 2), likely because of the deprotonation of the functional groups on the stationary phase and analyte. Notably, the capacity factor for the aforementioned analytes was observed to be very different; these analytes are usually retained to a lesser extent and thus eluted quickly. However, these analytes also had greater resolution even on the small retention scale. We also observed improvements in capacity factors, resulting in the enhancement of the resolution factor, similar to the case of enrofloxacin and difloxacin. Apparently, the enhancement of dipole-dipole interactions under the predominantly acidic additive was not generally advantageous to the resolution of fluoroquinolone-based antibiotics on the phenylethyl-bonded phase under nonaqueous elution.

Except for manipulating the capacity factor of analytes and avoiding possible hydrolysis of the stationary phase under nonaqueous conditions, it is possible to selectively reverse the elution order of some fluoroquinolone-based antibiotics and their precursors on the phenylethyl-bonded phase. This could be accomplished by adjusting rather than enhancing the degree of dipole-dipole interactions by altering the volume of acidic or basic additive in the mobile phase. Table 1 shows that the elution order of nalidixic acid and cinoxacin was reversed as the volume of acidic additive in the mobile phase was increased to 4 ml or higher. Elution reversal was also observed in ofloxacin and lomefloxacin when the volume of acidic additive in the mobile phase was 5 ml. Table 2 shows that the reversed elution of nalidixic acid and cinoxacin was observed when the volume of basic additive in the mobile phase was increased to 4 ml. Notably, by switching the mobile phase from 490/10/1/2 by volume (acetonitrile/methanol/acetic acid/triethylamine, *v*/*v*) to 490/10/2/1, the elution of enrofloxacin and difloxacin was reversed. Upon close examination of the structure of these analytes, each analyte contained a tertiary amine attached to the moiety suitable for the creation of steric hindrance. However, some analytes such as lomefloxacin, ciprofloxacin, and pipemidic acid bore secondary amines for retention purposes only.

## 4. Conclusion

Under nonaqueous elution, the resolution of 10 fluoroquinolone-based antibiotics and their precursors on the phenylethyl-bonded phase was isocratically, rapidly, and efficiently performed within 10 minutes. Based on the interaction simulation and FTIR data, *π*-*π* complexation and hydrophobicity were not the major forces contributing to this resolution. Instead, strong interactions between residual silanol groups on the silica gel were responsible.

Manipulating the capacity factor of analytes by altering the volume of acidic or basic additive in the mobile phase can reverse the elution order and improve the selectivity factor, thereby considerably enhancing the resolution factor of the analytes in some cases. These results would be very useful in determining these antibiotics in the dairy products under nonaqueous elution, which has been currently under investigation. In any case, avoiding possible hydrolysis of the stationary phase under nonaqueous elution conditions could be expected.

## Figures and Tables

**Table 1:** Chromatographic data for 9 quinolone-based antibiotics and their precursors under acidic nonaqueous elution on the phenylethyl-bonded stationary phase.

Compound^a^	NA (6)	CIN (8)	DIFL (10)	ENR (3)	PEFL (4)	OFL (1)	LOM (9)	CIP (2)	PA (5)
Structure	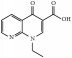	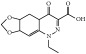	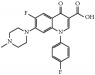	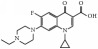	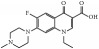	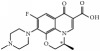	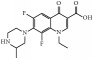	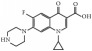	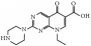
490/10/1/1	$\alpha =1.28\;\;{\left(\frac{{k^{\prime }}_{CIN}}{{k^{\prime }}_{NA}}=\frac{0.23}{0.18}\right)}^{b}$		*α* = 1.00 (*k*′_DIFL_ = *k*′_ENR_ = 0.72)		$\alpha =1.30\;\;\left(\frac{{k^{\prime }}_{OFL}}{k_{PEFL}}=\frac{1.31}{1.01}\right)$; $\alpha =1.24\;\;\left(\frac{{k^{\prime }}_{LOM}}{{k^{\prime }}_{OFL}}=\frac{1.63}{1.31}\right)$			$\alpha =1.18\;\;\left(\frac{{k^{\prime }}_{PA}}{{k^{\prime }}_{CIP}}=\frac{2.89}{2.46}\right)$	
490/10/1.5/1	*α* = 1.00 (*k*′_CIN_ = *k*′_NA_ = 0.18)		*α* = 1.00 (*k*′_DIFL_ = *k*′_ENR_ = 0.82)		$\alpha =1.33\;\;\left(\frac{{k^{\prime }}_{OFL}}{{k^{\prime }}_{PEFL}}=\frac{1.55}{1.17}\right)$; $\alpha =1.21\;\;\left(\frac{{k^{\prime }}_{LOM}}{{k^{\prime }}_{OFL}}=\frac{1.87}{1.55}\right)$			$\alpha =1.14\;\;\left(\frac{{k^{\prime }}_{PA}}{{k^{\prime }}_{CIP}}=\frac{3.17}{2.78}\right)$	
490/10/2/1	*α* = 1.00 (*k*′_CIN_ = *k*′_NA_ = 0.15)		$\alpha =1.10\;\;\left(\frac{{k^{\prime }}_{ENR}}{{k^{\prime }}_{DIFL}}=\frac{0.94}{0.85}\right)$; *R*_*s*_ = 0.69		$\alpha =1.36\;\;\left(\frac{{k^{\prime }}_{OFL}}{{k^{\prime }}_{PEFL}}=\frac{1.70}{1.25}\right)$; $\alpha =1.15\;\;\left(\frac{{k^{\prime }}_{LOM}}{{k^{\prime }}_{OFL}}=\frac{1.95}{1.70}\right)$			$\alpha =1.13\;\;\left(\frac{{k^{\prime }}_{PA}}{{k^{\prime }}_{CIP}}=\frac{3.14}{2.78}\right)$	
490/10/3/1	*α* = 1.00 (*k*′_CIN_ = *k*′_NA_ = 0.15)		$\alpha =1.21\;\;\left(\frac{{k^{\prime }}_{ENR}}{{k^{\prime }}_{DIFL}}=\frac{1.11}{0.92}\right)$; *R*_*s*_ = 1.24		$\alpha =1.38\left(\frac{{k^{\prime }}_{OFL}}{{k^{\prime }}_{PEFL}}=\frac{1.98}{1.40}\right)$; $\alpha =1.05\;\;\left(\frac{{k^{\prime }}_{LOM}}{{k^{\prime }}_{OFL}}=\frac{2.04}{1.94}\right)$			$\alpha =1.11\;\;\left(\frac{{k^{\prime }}_{PA}}{{k^{\prime }}_{CIP}}=\frac{3.10}{2.78}\right)$	
490/10/4/1	$\alpha =1.73\;\;{\left(\frac{{k^{\prime }}_{NA}}{{k^{\prime }}_{CIN}}=\frac{0.26}{0.15}\right)}^{c}$		$\alpha =1.30\;\;\left(\frac{{k^{\prime }}_{ENR}}{{k^{\prime }}_{DIFL}}=\frac{1.22}{0.94}\right);\;\;R_{s}=5.53$		$\alpha =1.35\;\;\left(\frac{{k^{\prime }}_{OFL}}{{k^{\prime }}_{PEFL}}=\frac{1.98}{1.46}\right)$; *α* = 1.00 (*k*′_LOM_ = *k*′_OFL_ = 1.98)			$\alpha =1.09\;\;\left(\frac{{k^{\prime }}_{PA}}{{k^{\prime }}_{CIP}}=\frac{2.88}{2.64}\right)$	
490/10/5/1	$\alpha =1.25\;\;{\left(\frac{{k^{\prime }}_{NA}}{{k^{\prime }}_{CIN}}=\frac{0.15}{0.12}\right)}^{c}$		$\alpha =1.38\;\;\left(\frac{{k^{\prime }}_{ENR}}{{k^{\prime }}_{DIFL}}=\frac{1.32}{0.96}\right)$; *R*_*s*_ = 7.15		$\alpha =1.29\;\;\left(\frac{{k^{\prime }}_{OFL}}{{k^{\prime }}_{PEFL}}=\frac{1.95}{1.51}\right)$; $\alpha =1.03\;\;{\left(\frac{{k^{\prime }}_{OFL}}{{k^{\prime }}_{LOM}}=\frac{1.95}{1.89}\right)}^{c}$			*α* = 1.00 (*k*′_PA_ = *k*′_CIP_ = 2.68)	

^a^The quinolone-based compounds are listed, from left to right, in the same order as that eluted with 490/10/2/1 mobile phase on the phenylethyl-bonded stationary phase. NA, CIN, DIFL, ENR, PEFL, OFL, LOM, CIP, and PA are abbreviations for nalidixic acid, cinoxacin, difloxacin, enrofloxacin, pefloxacin, ofloxacin, lomefloxacin, ciprofloxacin, and pipemidic acid, respectively.  ^b^The capacity and selectivity factors are calculated according to the equations *k*′ = (*t*_*r*_ − *t*_0_)/*t*_0_, *α* = *k*′_2_/*k*′_1_, and *R*_*s*_ = 2(*t*_*r*2_ − *t*_*r*1_)/(*w*_1_ + *w*_2_). The void volume of the column, *t*_0_, is 2.96 minutes. *α* = 1.00 means no separation.  ^c^The elution order is reversed, as compared to that with 490/10/2/1 mobile phase described above. An optically active carbon is associated with an asterisk for distinction.

**Table 2:** Chromatographic data for 9 quinolone-based antibiotics and their precursors under basic nonaqueous elution on the phenylethyl-bonded stationary phase.

Compound^a^	NA (6)	CIN (8)	ENR (3)	DIFL (10)	PEFL (4)	OFL (1)	LOM (9)	CIP (2)	PA (5)
Structure	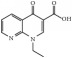	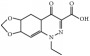	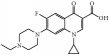	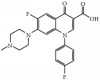	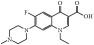	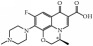	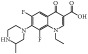	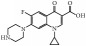	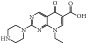
490/10/1/0.5	*α* = 1.00 (*k*′_CIN_ = *k*′_NA_ = 0.18)^b^		*α* = 1.00 (*k*′_DIFL_ = *k*′_ENR_ = 0.73)		$\alpha =1.38\;\;\left(\frac{{k^{\prime }}_{OFL}}{{k^{\prime }}_{PEFL}}=\frac{1.71}{1.24}\right)$; $\alpha =1.20\;\;\left(\frac{{k^{\prime }}_{LOM}}{{k^{\prime }}_{OFL}}=\frac{2.05}{1.71}\right)$			$\alpha =1.11\;\;\left(\frac{{k^{\prime }}_{PA}}{{k^{\prime }}_{CIP}}=\frac{3.36}{3.02}\right)$	
490/10/1/1	$\alpha =1.28\;\;\left(\frac{{k^{\prime }}_{CIN}}{{k^{\prime }}_{NA}}=\frac{0.23}{0.18}\right)$		*α* = 1.00 (*k*′_DIFL_ = *k*′_ENR_ = 0.72)		$\alpha =1.30\;\;\left(\frac{{k^{\prime }}_{OFL}}{{k^{\prime }}_{PEFL}}=\frac{1.31}{1.01}\right)$; $\alpha =1.24\;\;\left(\frac{{k^{\prime }}_{LOM}}{{k^{\prime }}_{OFL}}=\frac{1.63}{1.31}\right)$			$\alpha =1.18\;\;\left(\frac{{k^{\prime }}_{PA}}{{k^{\prime }}_{\;}}=\frac{2.89}{2.46}\right)$	
490/10/1/1.5	$\alpha =1.56\;\;\left(\frac{{k^{\prime }}_{CIN}}{{k^{\prime }}_{NA}}=\frac{0.28}{0.18}\right)$		$\alpha =1.07\;\;\left(\frac{{k^{\prime }}_{DIFL}}{{k^{\prime }}_{ENR}}=\frac{0.60}{0.56}\right)$; *R*_*s*_ = 0.64		$\alpha =1.24\;\;\left(\frac{{k^{\prime }}_{OFL}}{{k^{\prime }}_{PEFL}}=\frac{1.03}{0.83}\right)$; $\alpha =1.30\;\;\left(\frac{{k^{\prime }}_{LOM}}{{k^{\prime }}_{OFL}}=\frac{1.34}{1.03}\right)$			$\alpha =1.23\;\;\left(\frac{{k^{\prime }}_{PA}}{{k^{\prime }}_{CIP}}=\frac{2.55}{2.07}\right)$	
490/10/1/2	$\alpha =1.76\;\;\left(\frac{{k^{\prime }}_{CIN}}{{k^{\prime }}_{NA}}=\frac{0.32}{0.18}\right)$		$\alpha =1.13\;\;\left(\frac{{k^{\prime }}_{DIFL}}{{k^{\prime }}_{ENR}}=\frac{0.55}{0.49}\right)$; *R*_*s*_ = 0.75		$\alpha =1.24\;\;\left(\frac{{k^{\prime }}_{OFL}}{{k^{\prime }}_{PEFL}}=\frac{0.88}{0.71}\right)$; $\alpha =1.33\;\;\left(\frac{{k^{\prime }}_{LOM}}{{k^{\prime }}_{OFL}}=\frac{1.17}{0.88}\right)$			$\alpha =1.26\;\;\left(\frac{{k^{\prime }}_{PA}}{{k^{\prime }}_{CIP}}=\frac{2.33}{1.85}\right)$	
490/10/1/4	$\alpha =2.28\;\;{\left(\frac{{k^{\prime }}_{NA}}{{k^{\prime }}_{CIN}}=\frac{0.41}{0.18}\right)}^{c}$		$\alpha =1.12\;\;\left(\frac{{k^{\prime }}_{DIFL}}{{k^{\prime }}_{ENR}}=\frac{0.46}{0.41}\right)$; *R*_*s*_ = 0.93		$\alpha =1.22\;\;\left(\frac{{k^{\prime }}_{OFL}}{{k^{\prime }}_{PEFL}}=\frac{0.67}{0.55}\right)$; $\alpha =1.43\left(\frac{{k^{\prime }}_{LOM}}{{k^{\prime }}_{OFL}}=\frac{0.96}{0.67}\right)$			$\alpha =1.35\;\;\left(\frac{{k^{\prime }}_{PA}}{{k^{\prime }}_{CIP}}=\frac{2.07}{1.53}\right)$	

^a^The quinolone-based compounds are listed, from left to right, in the same order as that eluted with 490/10/1/2 mobile phase on the phenylethyl-bonded stationary phase shown in Figure 1. NA, CIN, ENR, DIFL, PEFL, OFL, LOM, CIP, and PA are abbreviations for nalidixic acid, cinoxacin, enrofloxacin, difloxacin, pefloxacin, ofloxacin, lomefloxacin, ciprofloxacin, and pipemidic acid, respectively. ^b^The capacity and selectivity factors are calculated according to the equations *k*′ = (*t*_*r*_ − *t*_0_)/*t*_0_ and *α* = *k*′_2_/*k*′_1_. The *t*_0_ value of the column is 2.96 minutes. *α* = 1.00 means no separation. ^c^The elution order is reversed, as compared to that with 490/10/1/2 mobile phase described above. An optically active carbon is associated with an asterisk for distinction.

**Table 3:** Theoretical interaction simulation results between 6 selected antibiotics and 3 stationary phase modifiers.

	Phenyl			Phenylethyl			Phenylhexyl		
	*X*(*E*_min_ kJ/mol)^a^	*x*≦*x* + 4^b^	*x* + 4 < *x*≦*x* + 13	*X*(*E*_min_ kJ/mol)	*x*≦*x* + 4	*x* + 4 < *x*≦*x* + 13	*X*(*E*_min_ kJ/mol)	*x*≦*x* + 4	*x* + 4 < *x*≦*x* + 13
Difloxacin	−421.94	5	3	−487.17	6	6	−579.45	7	15
Enrofloxacin	−287.17	6	4	−354.32	3	8	−439.95	9	10
Ofloxacin	−517.35	4	3	−575.98	5	8	−676.15	3	16
Pipemidic acid	−168.29	6	3	−233.93	5	5	−315.88	5	15
Cinoxacin	−426.14	3	2	−483.279	4	10	−575.23	7	10
Nalidixic acid	−227.30	4	2	−294.71	6	5	−383.05	6	10

^a^The analyte and the modifier of stationary phase are first minimized in energy before placing the modifier around the analyte to calculate the energy released upon interaction based on the semiempirical method. These collected energy values are then categorized into the defined intervals. ^b^The number of the released energy values that fall in the interval.

## Additional Points

Under the same nonaqueous chromatographic conditions, the elution order was reversed as switching the stationary phase from phenylethyl- to C_18_-based. The elution reversal was also observed as altering the acid/base ratio in the mobile phase.

## Supplementary Materials

Supplementary Materials The interaction simulation after energy minimization between ofloxacin and C_18_ moiety expressed in stereochemistry molecular (left) and stick and ball (right) models for easy comparison. The hydrophobic interaction is clearly dominated in the simulation.
