# Supplementary material for: Isocratic Resolution of Fluoroquinolone-Based Antibiotics on the Phenylethyl-Bonded Phase under Nonaqueous Elution: A Consideration of the Separation Mechanism
Source: Int J Anal Chem. 2018 May 31;2018:1375215. doi: 10.1155/2018/1375215 (PMC6000864; doi:10.1155/2018/1375215)
Supplement: Supplementary Materials — The interaction simulation after energy minimization between ofloxacin and C18 moiety expressed in stereochemistry molecular (left) and stick and ball (right) models for easy comparison. The hydrophobic interaction is clearly dominated in the simulation. [file 1375215.f1.docx]

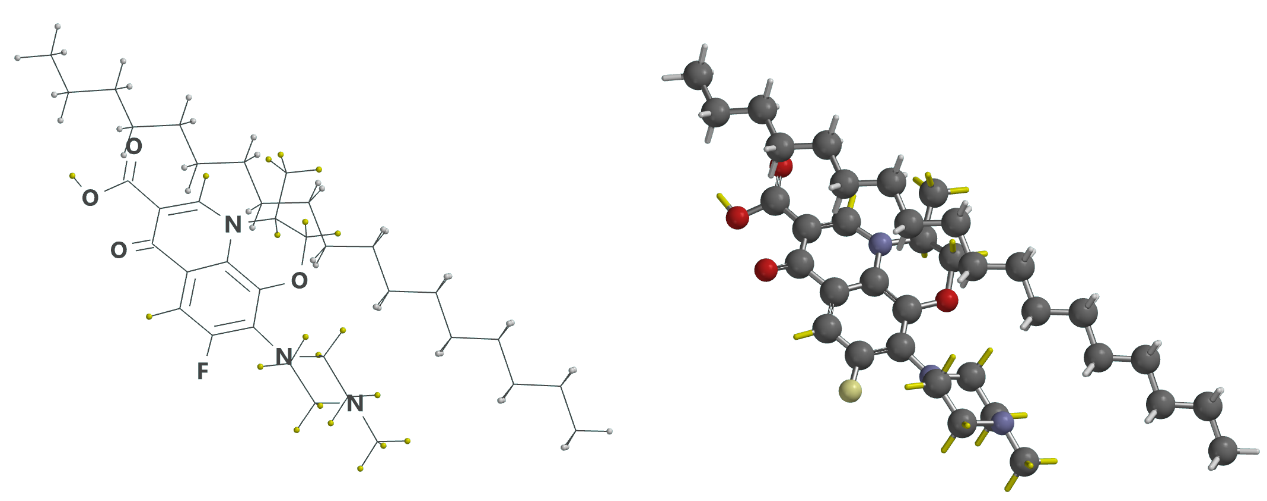


The interaction simulation after energy minimization between ofloxacin and C_18_ moiety expressed in stereochemistry molecular (left), stick and ball (right) models for easy comparison. The hydrophobic interaction is clearly dominated in the simulation.
